# Identification and Characterization of miRNA Transcriptome in Asiatic Cotton (*Gossypium arboreum*) Using High Throughput Sequencing

**DOI:** 10.3389/fpls.2017.00969

**Published:** 2017-06-15

**Authors:** Muhammad Farooq, Shahid Mansoor, Hui Guo, Imran Amin, Peng W. Chee, M. Kamran Azim, Andrew H. Paterson

**Affiliations:** ^1^Molecular Virology and Gene Silencing Laboratory, Agricultural Biotechnology Division, National Institute for Biotechnology and Genetic EngineeringFaisalabad, Pakistan; ^2^Plant Genome Mapping Laboratory, University of Georgia, AthensGA, United States; ^3^Jamil-ur-Rahman Center for Genome Research, International Center for Chemical and Biological Sciences, University of KarachiKarachi, Pakistan

**Keywords:** microRNA, *Gossypium arboreum*, transcriptome, bioinformatics, next generation sequencing

## Abstract

MicroRNAs (miRNAs) are small 20–24nt molecules that have been well studied over the past decade due to their important regulatory roles in different cellular processes. The mature sequences are more conserved across vast phylogenetic scales than their precursors and some are conserved within entire kingdoms, hence, their loci and function can be predicted by homology searches. Different studies have been performed to elucidate miRNAs using *de novo* prediction methods but due to complex regulatory mechanisms or false positive *in silico* predictions, not all of them express in reality and sometimes computationally predicted mature transcripts differ from the actual expressed ones. With the availability of a complete genome sequence of *Gossypium arboreum*, it is important to annotate the genome for both coding and non-coding regions using high confidence transcript evidence, for this cotton species that is highly resistant to various biotic and abiotic stresses. Here we have analyzed the small RNA transcriptome of *G. arboreum* leaves and provided genome annotation of miRNAs with evidence from miRNA/miRNA^∗^ transcripts. A total of 446 miRNAs clustered into 224 miRNA families were found, among which 48 families are conserved in other plants and 176 are novel. Four short RNA libraries were used to shortlist best predictions based on high reads per million. The size, origin, copy numbers and transcript depth of all miRNAs along with their isoforms and targets has been reported. The highest gene copy number was observed for gar-miR7504 followed by gar-miR166, gar-miR8771, gar-miR156, and gar-miR7484. Altogether, 1274 target genes were found in *G. arboreum* that are enriched for 216 KEGG pathways. The resultant genomic annotations are provided in UCSC, BED format.

## Introduction

Cotton (genus *Gossypium;* family *Malvaceae*) having approximately 50 species (Wendel and Cronn, 2003; Wendel et al., 2009), is an important cash crop with cumulative worldwide economic impact estimated to be approximately 500 billion USD per year in terms of textile production, jobs and food products (Yuan et al., 2015). Four species, *Gossypium hirsutum*, *G. barbadense, G. arboreum*, and *G. herbaceum* have been widely cultivated for their fiber and oil. *G. hirsutum* (AADD, AD1; 2*n* = 52) accounts for approximately 90% of worldwide cotton production (Li et al., 2015; Liu et al., 2016) due to its better yield and good fiber quality, whereas *G. barbadense* (AADD, AD2; 2*n* = 52), also known as sea-island cotton, is valued for its superior fiber length, strength and fineness (Yuan et al., 2015). *G. arboreum* (AA, 2*n* = 26), also known as Desi cotton or Asiatic cotton, is another important cultivated cotton species and a putative contributor of the A sub-genome and the maternal parent of tetraploid cotton species (Li et al., 2014). *G. raimondii*, which is a diploid (DD, 2*n* = 26) cotton species, is not cultivated but is thought to closely resemble the pollen-providing contributor of the D sub-genome to *G. hirsutum* and *G. barbadense* in an allopolyploidization event occurred approx. 1–2 million years ago (Cronn et al., 1999; Wang K. et al., 2012).

The cotton industry is facing yield losses due to several biotic, e.g., cotton leaf curl disease (CLCuD) (Briddon, 2000) and abiotic stresses including temperature, salinity, drought and cold (Padmalatha et al., 2012). The cumulative reduction in cotton yield only because of insects was up to 7.66% in the United States in 1999 (Williams et al., 2000) and up to 5 billion USD from 1992 to 1997 in Pakistan (Briddon, 2000) due to CLCuD. According to a 2007 Food and Agriculture Organization (FAO) report, soil waterlogging affected 20–30 million hectares of irrigated land area due to highly changing weather condition (Najeeb et al., 2015). *G. hirsutum* is susceptible to these stresses (Mahmood et al., 2003), whereas *G. arboreum* is inherently tolerant but having poor fiber quality (Ahmad et al., 2011). Introgression of resistance from Asiatic cotton to upland cotton (*G. hirsutum*) has been applied through conventional breeding techniques (Ahmad et al., 2011), thus it is important to fully understand and characterize the resistance mechanisms in diploid progenitors, for engineering of higher resistance in economically important cotton species using molecular techniques. The complete genome annotation of both coding and especially non-coding regulatory components including microRNAs (miRNA), small nuclear RNAs (snRNA), small nucleolar RNAs (snoRNAs), ribosomal RNAs (rRNAs), transfer RNAs (tRNAs) and the repetitive elements, of a *G. arboreum* draft genome, available since 2014 (Li et al., 2014), may help to elucidate its superior transcriptional and post-transcriptional expression regulation mechanisms for higher resistance.

Small endogenous RNAs are of two types in plants (Axtell and Bowman, 2008; Wang Z.-M. et al., 2012): small interfering RNAs (siRNAs) and miRNAs. Endogenous siRNAs are further classified into repeat-associated siRNAs (rasiRNAs), *trans-acting* siRNAs (tasiRNAs) and natural antisense siRNAs (nat-siRNAs). The various endogenous miRNAs contribute by similar mechanisms to post-transcriptional gene regulation but they greatly differ in biogenesis from siRNAs because they have well characterized genomic origins. Some miRNAs originated from independent well characterized genes and some are encoded by introns of other protein coding genes. Moreover, retrotransposons have also been found to be an origin, hence copy number variations due to gene loss and gain is also observed during miRNA evolution (Li and Mao, 2007; Axtell and Bowman, 2008; Voinnet, 2009; Cuperus et al., 2011). The miRNAs are small 20–24nt molecules that have significant regulatory role in different cellular processes including transcription regulation (Fazi et al., 2005), development (Xie et al., 2015), intercellular signaling (Curaba et al., 2014), biotic and abiotic stress response (Khraiwesh et al., 2012), cell development life cycle control and pathogenicity response (Sunkar et al., 2012), by negatively regulating their target genes. Protein coding genes are not the only targets of miRNAs; instead, several other non-coding RNAs including tasiRNAs (Xie et al., 2015) and competing endogenous RNAs (ceRNAs) (Le et al., 2016), are also regulated by these small molecules. Therefore, it is important to identify and characterize the miRNAome of sRNAs for this important cotton species.

Plant miRNA precursors have an average size of 70nt and are derived from primary miRNAs (pri-miRNAs) by the action of endoribonuclease III Drosha, whereas mature sequences are obtained from precursors after cutting using DICER-LIKE1 (Motameny et al., 2010). Both mature miRNA and miRNA^∗^ may co-regulate gene expression (Luo et al., 2012) or sometimes miRNA^∗^ behaves like miRNA as well (Motameny et al., 2010), but often mature miRNA sequence is responsible for transcriptional (Chen, 2009; Wang Z.-M. et al., 2012) level by chromatin remodeling (Axtell and Bowman, 2008) and (or) post-transcriptional gene silencing (PTGS) using an RNA induced silencing complex (RISC) (Xue et al., 2013). The level of conservation in mature sequences, especially within the seed region, is extremely high across different species when compared with their precursors (Axtell and Bowman, 2008). Under these circumstances homology searches can play a useful tool to predict their loci and functions (Zhang et al., 2006a). These small RNAs (sRNAs) build another layer of complexity in gene expression control within genomic network (Zhang et al., 2013).

Many reports have discussed the prediction of miRNAs with good precision and accuracy within the short RNA (sRNA) transcriptome using High Throughput Sequencing (HTS) and subsequent bioinformatics analysis, confirmed using wet-lab experiments (Padmalatha et al., 2012; Wang Z.-M. et al., 2012; Xue et al., 2013; Zhang et al., 2013; Wang et al., 2016). To date, 80 miRNAs have been identified in *G. hirsutum*, 296 in *G. raimondii*, and only 01 in both *G. herbaceum* and *G. arboreum* (Griffiths-Jones et al., 2006). To our knowledge, this will be the first report on identification of miRNAs from the *G. arboreum* sRNA transcriptome. In this study, we have prepared four different sRNA libraries from *G. arboreum* leaves, sequenced these using the illumina GA-II next generation sequencing (NGS) platform and identified and characterized the transcriptome using bioinformatics tools. The results suggest with good confidence that HTS can be used along with bioinformatics tools for accurate prediction of miRNA genes at the genome scale.

## Materials and Methods

### Plant Materials and Growth Conditions

*Gossypium arboreum* seeds were kindly provided by Prof. Paterson. Plants were sown under control, drought, salinity and heat conditions in green house or growth chamber. For salinity stress plants at 4–5 leaf stage were watered with 200 mM sodium chloride solution for 1 week and leaves were collected. For drought plants at 4–5 leaves growing in pots were not watered for a week and leaves were collected. For heat stress plants were grown in a growth chamber at 50^o^ for 1 week and leaves were collected. These different stresses were used to identify treatment specific transcripts, which are not expressed in one condition. The purpose here was to identify maximum number of miRNA but the role of miRNAs induced under a particular condition is the subject of another manuscript. Total RNA was extracted and samples were sent to LC Sciences^®^ United States^1^ for sequencing of sRNA using NGS.

### Library Preparation for NGS

Single-end sequencing of sRNA was conducted on Illumina GA-II NGS platform. The Illumina TruSeq sRNA kit as used to prepare the library and size selection of 145–160nt was performed after ligation. Confirmation of RNA sample concentration was performed on a nanodrop spectrophotometer and RNA quality on an Agilent BioAnalyzer.

### Reference Datasets

The reference genome was obtained from the Cotton Genome Project website^2^, assembled by Beijing Genomic Institute (BGI), release1.0 (Li et al., 2014). We used miRBase release 21 (Griffiths-Jones et al., 2006) for plants miRNA mature and stem-loops sequence reference using linux command line. For filtering of non-coding RNAs other than miRNAs and other RNA structural families, we have used Rfam release 12.0 (Nawrocki et al., 2014). The sequence data was deposited into NCBI public databases under following accession; SRA: SRP095309, BioProject: PRJNA357977 and BioSamples: SAMN06163076, SAMN06163077, SAMN06163078, and SAMN06163079.

### Preprocessing

The sRNA deep sequencing data retrieved from Next Generation Sequencer was preprocessed to remove adaptor sequences and quality filtering using Cutadapt *v1.1* (Martin, 2011) and validated using CLC Genomics Workbench *v7.5* as well. Both adaptors were removed whereas the some portion of 3′ adaptor “TGGAATTCTCGGGTGCCAAGG” was still present in 36nt raw sRNA reads as the actual payload was 20–24 bp longer (Martin, 2011). The first 6 bases (TGGAAT) were mapped to raw reads to locate their position and the adaptor is then trimmed from the raw reads, e.g., a raw read of TCCATTGTCGTCCAGTGGTTAGGATATGGAATTCTC is trimmed to a read of TCCATTGTCGTCCAGTGGTTAGGATA. Reads were filtered out if the adaptor cannot map to the raw reads or length of the read is less than 15nt after trimming.

### Filtering Using Rfam and Uniprot

We used local blastn *v2.2.26* tool from National Center for Biotechnology Information (NCBI) for searching in Rfam *v12.0* database using word size of 11. Reads with zero mismatches, zero gaps, 100% identity and 100% query coverage were removed from miRNAome. Similarly, the NCBI blastx tool was used to search in uniprot protein databases using default word size and the same criteria were used to filter out possible messenger RNA (mRNA) residues.

### Prediction Pipeline

To predict miRNAs from small RNA-Seq data, using expression patterns and following the criteria for plant miRNA annotation, miR-PREFer (Lei and Sun, 2014) was used due to its low false-positive rate and running time. The mappable reads were aligned to the *G. arboreum* reference genome using bowtie2 (Langmead and Salzberg, 2012) with zero mismatch and end to end read mapping and multiple mappings allowed. The resultant SAM file was fed into miR-PREFer pipeline along with *G. arboreum* CDS annotation to exclude all predictions within exons. The precursor’s length was set to 150 and reads depth cutoff was set to 5. The minimum mature sequence length range was set to 18 to 24 with no star sequence expression allowed. The resultant candidate precursors were checked against miRNA primary and ancillary annotation criteria (Meyers et al., 2008). Precursor secondary structures was predicted using RNALfold (Lorenz et al., 2011) and minimum free energy index (MFEI) (Zhang et al., 2006b) was selected as less than or equal to -0.85 and necessary downstream analysis was performed using shell and perl scripting under Centos 6.5 Linux environment.

### Identification of miRNA Families in *G. arboreum*

The predicted candidate precursors were clustered into miRNA families using Cd-hit v4.6.1 (Li and Godzik, 2006) using sequence identity cut-off 0.9 and bandwidth of alignment set to 20. To identify conserved miRNAs, we used blastn to match the 8496 mirBase release 21 from 73 plant families with not more than 3 nucleotide difference, i.e., no more than 3 mismatches with zero gaps in mature sequences. The best match was selected if multiple hits are found to satisfy matching criteria. For partial matches, we’ve analyzed the flanking regions matching the previously known sequences and if the matching criterion is satisfied, then that candidate is assigned that particular family.

### Genomic Origins of miRNAs

To elucidate the origin of miRNAs and validate the ancillary criteria of miRNA annotations (Meyers et al., 2008), overlapped miRNA positions were checked using transcripts annotations of the genome. The annotations were converted to UCSC bed file format and their overlaps were predicted using bedtools (Quinlan and Hall, 2010). Hence the intronic and intergenic origins were calculated but miRNAs of repetitive origin are still unknown due to unavailability of repeat annotation. The positional clusters of miRNA genes were found using *cluster* tool from bedtools using strand specific (-s) and window size (-d) of 10000.

### MiRNA Validation

RNA was isolated from different tissues of *G. arboreum* using total plant RNA purification reagent (Invitrogen Cat No. 12322-012). RNA samples (30 μg) were diluted in 2X loading dye (95% formamide, 0.025% xylene cyanol, 0.025% bromophenol blue and 5mM EDTA) incubated at 65°C for 10 min for denaturation and placing on ice. 300pMol primer (complimentary to the probe) was loaded as positive control. Denatured RNA samples were loaded to 15% PAGE (Urea) and run at 100 V in BIO-RAD Mini protean tetrasystem United States. The gel was stained with ethidium bromide and visualized under UV light once the run was finished. RNA was transferred to the nylon membrane (Amersham Hybond^TM^-N++, GE Healthcare) using semi dry blotter (Transblot SD BIO-RAD United States) at 15V for 1 h. RNA was cross linked to the membrane using UV Cross Linker (CL100o ultraviolet cross linker, UVP). The DNA Probe was synthesized using DIG Oligonucleotide 3′-End Labeling Kit (2nd generation from Roche Life Science, Catalog No.3353575910). The blot was prehybridised at 42°C for 3h and hybridized with probe at 42°C for 16 h. The blot was washed with 2X SSC shortly then twice with 2xSSC/0.1% SDS for 15mins and 30mins at 60°C. The blot was developed by chromogenic (NBT/BCIP-T) method using supplied protocol (Amin et al., 2011; Zhang et al., 2013).

### Target Predictions

We used psRNATarget (Dai and Zhao, 2011) for miRNA target predictions against all *G. arboreum* annotated coding sequences from BGI, with maximum expectation value of 3.0 and hspsize equal to 20bp, allowed maximum energy to unpair the target site (UPE) equal to 25. Flanking length around target sites for target accessibility analysis was set at 17bp in upstream and 13bp in downstream. Range of central mismatch leading to translational inhibition was set from 9 to 11nt. The targets with more stringent miRNA-mRNA pairing were preferred; if multiple targets were found, all were reported as there may be multiple targets of a single miRNA with different specificity. The target genes were functionally annotated using the KEGG pathway database by the BlastKOALA sequence similarity tool^3^ and by alignment to the PANTHER v11.1 database of protein HMM models^4^ with the PANTHER scoring tool v2.0.

## Results

### Deep Sequencing of Small RNA Libraries of *G. arboreum*

**FIGURE 1:**
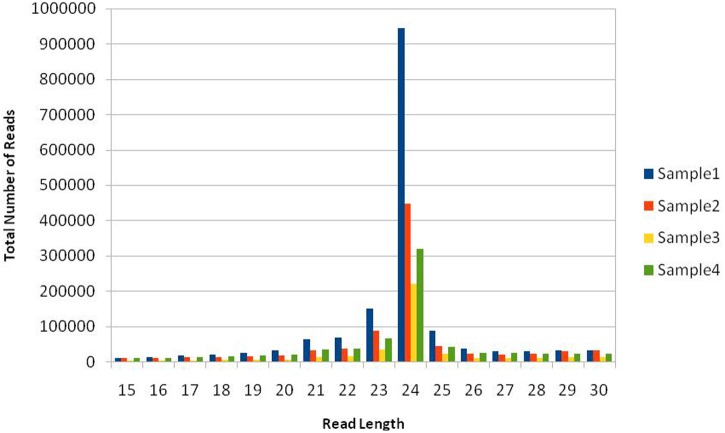
Size distribution of mappable short RNA (sRNA) reads. Sample1: Healthy, Sample2: Treated with artificial drought, Sample3: Treated with Salinity, Sample4: Treated with extra heat conditions. The histogram from deep sequencing short reads illustrates the distribution of sRNA read lengths in the dataset, suggesting different types of sRNAs, e.g., the peak at 21 belongs to siRNAand others signal about content of miRNAs either as complete or trimmed fragments.

Total read length obtained from NGS sequencer was 36nt after trimming everything above this size. This included both 5′ and 3′ adaptor sequences, but partial 3′ adaptor was still present due to smaller sRNA payload of 20–24nt, which was further removed. The reads in which 3′ adaptors were missing were most probably related to longer payloads and they were not considered as small RNAs –constituted approximately 58% of raw data from all libraries. This is in concordance with our previous understanding that plants have relatively large and complex small RNA populations within which miRNAs are often a minority (Meyers et al., 2008). We have obtained a total of 22.27 million potential sRNA reads out of which 7.64M, 5.095M, 4.635M, and 4.899M belong to four libraries, respectively. The raw reads were grouped into unique clusters, having a maximum of one ambiguous nucleotide, of 1.6M, 0.87M, 0.409M, and 0.724M reads for the four libraries, respectively. Total read length of 15 to 30 nucleotides was obtained after preprocessing including 3′ sequencing adaptor removal and quality filtering of raw reads, which were further mapped to the genome, known as mappable reads. The read quality on phred scale ranged from 24 to 40 and on average 39nt after this step, which increases the reliability of *in silico* prediction. The summary of raw data statistics is shown in **Table 1**. The read length distribution, as shown in **Figure 1**, suggests the distribution of different types of sRNAs in our libraries. A peak read length of ∼21 and 22 suggests that there is some content of sRNAs other than miRNAs (Meyers et al., 2008) present in our data and 22, 23 and 24 expresses the content of miRNAs, which is higher.

**Table 1:** Summary of short RNA raw NGS data.

	Sample1	Sample2	Sample3	Sample4
Total raw reads	7,640,730	5,095,200	4,635,233	4,899,320
Total unique clusters	1,608,017	870,343	409,940	724,817
Avg. read length after trimming	15–30	15–30	15–30	15–30
Average quality per read	39	39	39	39
Max N’s allowed per read	1	1	1	1
rRNA, tRNA, snRNA, and snoRNA (total raw reads count)	460,425	363,352	90441	279084
rRNA, tRNA, snRNA, and snoRNA (unique reads count)	61,919	59,294	24,255	58,744
Mapped to Uniprot (total reads cunt)	1071	849	290	1299
Mapped to Uniprot (unique reads count)	649	395	219	387

### Identification of miRNAs from *G. arboreum* miRNAome Using Bioinformatics

**FIGURE 2:**
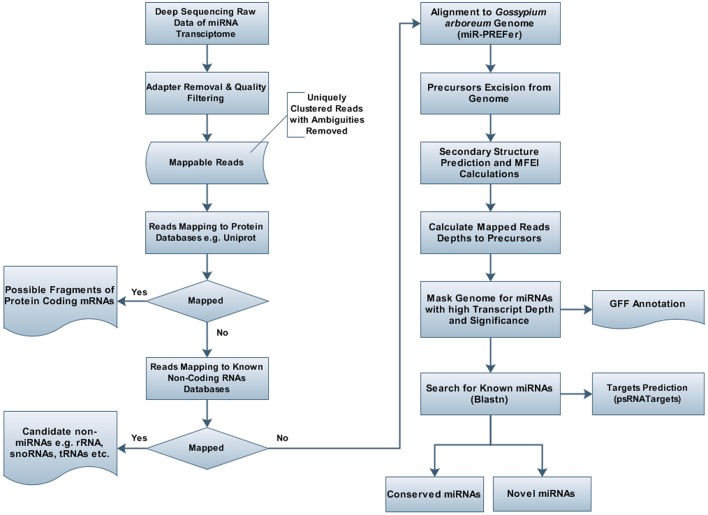
Flowchart for prediction of miRNAs and their targets in *Gossypium arboreum*. Deeply sequenced sRNA transcriptome of *G. arboreum* was mapped to different RNA databases, e.g., uniprot and rfam, after quality filtering and adapter trimming, to remove undesired RNA products. After obtaining purified candidate miRNA products, short reads were aligned to the whole genome and 250bp flanking regions were excised for candidate precursors, which were further analyzed by mIR-PREFer pipeline to obtain their miRBase mapping transcript depth, after fulfilling miRNA prediction criteria.

**FIGURE 3:**
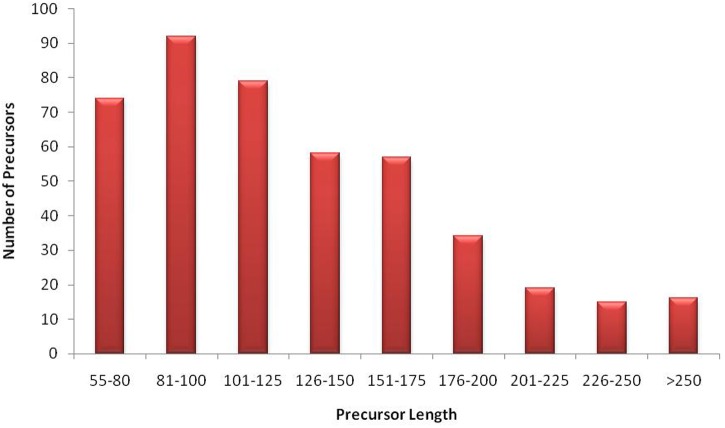
Size distribution of miRNA precursors. The graph illustrates that most of *G. arboreum* miRNA precursors are greater than 80nt long and less than 130nt. However, precursor sizes up to 300nt are also possible to exist.

**FIGURE 4:**
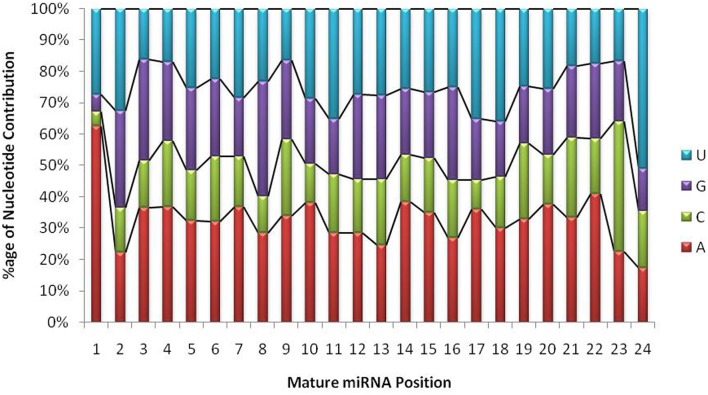
Position-wise nucleotide contributions in mature miRNA sequences. The graph shows that the first base in mature transcripts is either Adenine or Uracil with a probability of 0.9, whereas, the overall A+U composition ranges from 40 to 90% and G+C composition ranges from 10 to 61%. The probability of A/U is always greater than G/C throughout the mature transcript.

The unique raw mappable read clusters were mapped to uniprot protein databases, to filter all possible degraded protein coding mRNA products. The total reads mapped with perfect homology to uniprot are 1071 (0.014%), 849 (0.017%), 290 (0.006%), 1299 (0.026%) from libraries 1 to 4, respectively. Moreover, the mappable reads were searched in the RNA family (Rfam release 12.0) (Nawrocki et al., 2014) database to filter out other known non-coding RNA (tRNA, rRNA, snRNA, snoRNA, etc.) products and the total raw read count mapped were 460425 (6.02%), 363352 (7.13%), 90441 (1.95%), 279084 (5.7%) for the four libraries, respectively, as shown in **Table 1**. The uniprot and RFAM filtered data was mapped to the *G. arboreum* genome sequence (Li et al., 2014) and 1.19M (73.78%), 0.598M (68.72%), 0.298M (72.9%), and 0.452M (62.49%) of the perfectly matched reads were mapped at 4515822, 2332365, 1364987, and 1793749 different genomic locations from the four libraries, respectively. The alignment was obtained in Sequence Alignment Map (SAM) format and fed into miR-PREFer pipeline (Lei and Sun, 2014) for potential precursor prediction. The 250 base flanking regions were excised by considering both 5′ and 3′ mature sequence possibilities. The pipeline, when subjected to all libraries simultaneously, generated 948, 914, 924, and 914 potential precursors from the four libraries, respectively. These potential candidates were short listed based on total reads mapping depth to a precursor and presence in multiple libraries. The precursors with total mature transcript depth greater than 10 in at least one library were considered to be significant, as shown in Supplementary Table S1. The mappable reads were mapped to mature, star and antisense regions and positional and length variants were observed in different transcript mappings. This was because of varying DICER activity or data preprocessing artifacts. The miRNA/miRNA^∗^ duplex with positional variation less than 25% were selected for higher confidence (Meyers et al., 2008). Exact duplicates or overlapping predicted precursors were removed from potential precursors. The flowchart of complete data analysis has been shown in **Figure 2**. The precursor size ranged from 55 to 296nt with the majority within 101 to 150nt (30.8%), 55–80nt (16.7%), 81–100nt (20.7%), and 151–200nt (20.5%) and greater than 200 (11.3%) as shown in **Figure 3**. The mature sequences have A-U content greater than G-C throughout all mature transcripts except at position 23. Moreover, the total A-U content is greater than 50% in all positions except at position 9, which is immediately after seed region, as shown in **Figure 4**. The genomic coordinates of the predicted miRNAs are provided in UCSC, BED specification, see Supplementary File (*G.arboreum_miRNAs.bed*).

### Identification of Conserved and Non-conserved miRNAs

The miRNAs that are present in at least one major ancient clade of land plants are referred as ‘conserved’ (Axtell and Bowman, 2008) and those that are more likely to be evolved much more recently in our species of interest or has very limited phylogenetic distribution, are termed ‘novel’ or ‘species specific’ or ‘non-conserved.’ Altogether, 446 candidate precursors were shortlisted based on above-mentioned criteria and clustered into 224 miRNA families. Among the 224 families, 223 were present in more than one library. Candidate mature sequences mapped to 8496 mature miRNAs from 73 plants with no more than 3 nucleotide differences, i.e., sum of gaps and substitutions was taken to be less than 4nt, indicating 48 conserved and 176 *G. arboreum* specific or novel miRNAs as shown in **Table 2** (Voinnet, 2009). In particular, all 21 conserved miRNA families (Axtell and Bowman, 2008) (156, 159, 160, 162, 164, 166–169, 171, 172, 319, 390, 393–399, and 408) were found.

**Table 2:** MicroRNAs prediction and origin statistics.

	Conserved	*G. arboreum* specific (novel)	Total
Total miRNAs predicted	48	176	224
Total intronic miRNAs	8	11	19
Total intergenic miRNAs	40	165	205
Total miRNA genomic origins	188	258	446

### Target Gene Prediction

**FIGURE 5:**
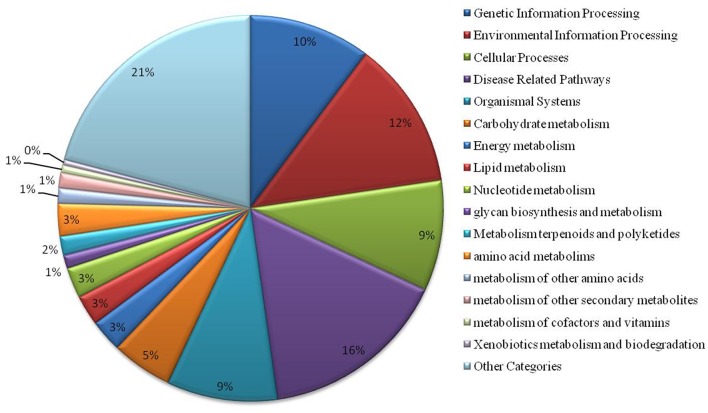
Functional characterization of miRNA targets in *G. arboreum* using KEGG. The chart illustrates different KEGG pathway groups, where most of the miRNAs target genetic information processing (10%), environmental signaling and response (12%), cellular processes (9%), disease related pathways (16%) and organismal systems (9%). There are several metabolism pathways as well as other KEGG pathway categories shown as aggregate value of 21% of the targets found and listed in Supplementary Table S3.

**FIGURE 6:**
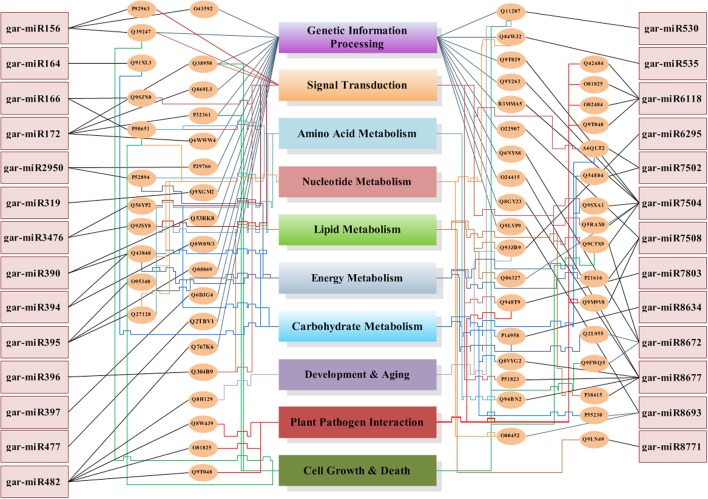
MicroRNA genes network. The miRNA genes network illustrates 27 miRNAs in rectangular boxes with their target uniprot proteins, elliptical boxes and connected to the miRNAs with straight lines in black color. These uniprot proteins are then attached to the biological processes, which they regulate using color coded lines. Each functional category has a unique colored line terminated into its box, starting from the elliptical protein id box. The lines with relatively higher weight illustrates that it is targeted from multiple protein boxes. The Genetic Information Processing box is targeted by straight inclined lines whereas all other categories have sharp edges lines.

**FIGURE 7:**
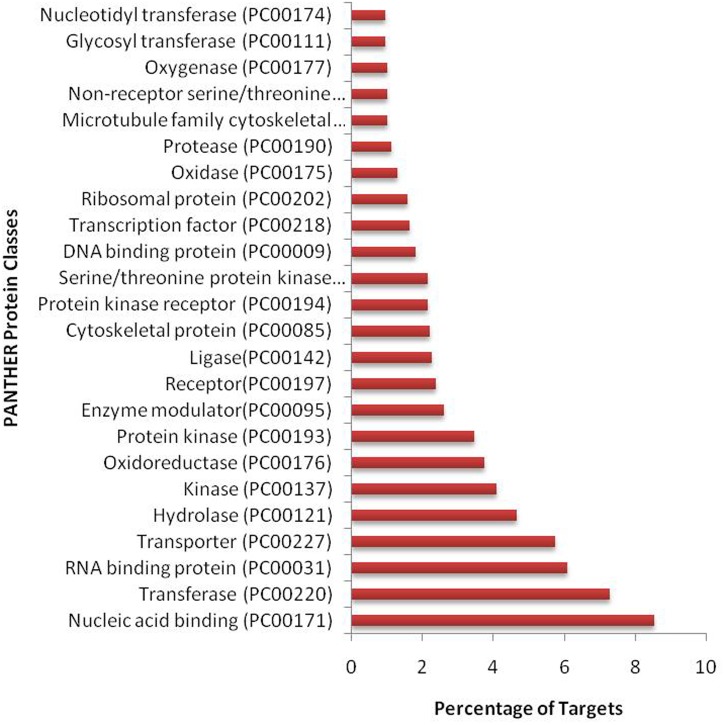
PANTHER protein classes of miRNA targets in *G. arboreum.* The vertical bar graph represents percentage of abundance of different protein classes from PANTHER^®^ database v10.0, in the target proteins. The most abundant target is DNA binding class, which includes transcription factors.

**FIGURE 8:**
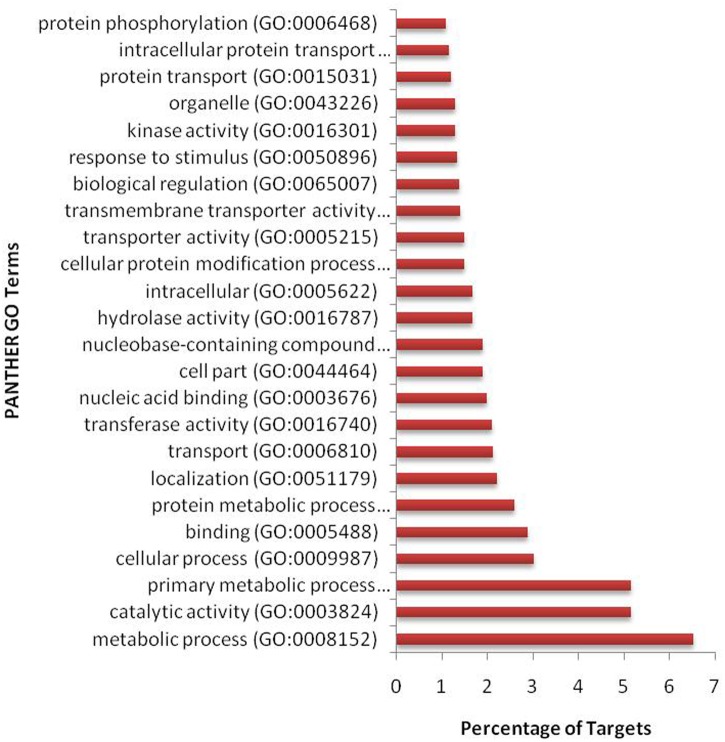
Gene ontology (GO) classification of miRNA targets in *G. arboreum*. The vertical bar graph represents percentage of abundance of different GO database terms from PANTHER^®^ database v10.0, in the target proteins.

We have found a total of 1274 targets for 206 miRNA families in *G. arboreum*, which complements the prediction reliability as the ancillary criteria of miRNA annotation (Meyers et al., 2008) and provided in Supplementary Table S2. The rest of 16 miRNAs may be either target-less elements that are evolutionarily transient or their targets are undiscovered due to lack of precision of target prediction tools. All of the conserved miRNAs do have their associated target genes but this is not true for all novel miRNAs. The highest number of target genes per miRNA was found to be 48 and average number of targets for conserved miRNAs (9) is larger than average number of targets for novels (4). The set of 1274 predicted target proteins were functionally characterized in the Kyoto Encyclopedia of Genes and Genomes (KEGG) pathway database^5^ by the BLASTKOALA (Kanehisa et al., 2015) sequence similarity tool. The BLASTKOALA annotated 441 (34.5%) out of 1274 to the KEGG orthologs database and 441 proteins were enriched in 216 different pathways (*TS3*). The distribution of these Kegg orthologs in different cellular functional categories has been shown in **Figure 5**. Altogether, 64 genes are involved in genetic information processing, whereas, other processes include environmental information processing (78), general cellular processes (58), disease related pathways (99), organismal systems (58) and metabolism of carbohydrates (32), energy (17), glycans (7), nucleotides (16), amino acids (25), cofactors and vitamins (4), lipids (16) and terpenoids and polyketides (10) and biosynthesis of other secondary metabolites (8). The rest were categorized in other pathways, as shown in *TS3*. Moreover, the 1274 target genes were annotated using PANTHER^®^ gene ontology and classification system (Mi et al., 2013). Altogether, 1184 out of 1274 target proteins were annotated to 614 protein families and 130 unique protein classes and 293 unique GO terms. The highest number of targets were related to DNA and nucleic acid binding proteins (150) and transferases (128) followed by RNA binding proteins (107), transporters (101), hydrolases (82), kinases (133), oxidoreductases (66) and enzyme modulators (46), etc. The miRNAs gene network, regulating different functional pathways, along with their target proteins in *G. arboreum* has been shown in **Figure 6**. The top 24 PANTHER^®^ classification having greater than 1% of target genes in terms of protein classes and GO terms are shown in **Figures 7**, **8**, respectively, whereas the rest of the targets are listed in Supplementary Table S4.

### Copy Numbers of miRNA Genes in *G. arboreum*

**FIGURE 9:**
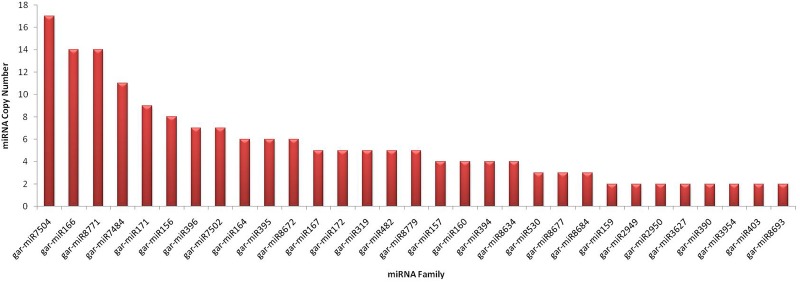
Conserved miRNA copy numbers in *G. arboreum*. The horizontal axis lists different conserved miRNA families with greater than or equal to two copies in the genome of *G. arboreum*. The vertical bars illustrate the exact copies of corresponding miRNA gene.

Varying miRNA copy numbers may affect target regulation capability of plants. These copy number variants (CNVs) precursors may or may not have exactly same nucleotide sequences, but their mature segments are more conserved. The gain or loss of miRNA genes is due to tandem and segmental duplication events (Li and Mao, 2007). Plants have relatively higher numbers of miRNA gene family members as compared to animals but smaller total numbers of miRNA genes. The average number of precursors in 224 genes was 2.0, which is in concordance with previous reports for plants (Li and Mao, 2007). The maximum copy number was observed for gar-miR7504 with 17 members and gar-miR166, gar-miR8771, gar-miR7484, gar-miR171, gar-miR156, gar-miR396, gar-miR7502, gar-miR164, gar-miR395, gar-miR8672 and gar-miR167 have 14, 14, 11, 9, 8, 7, 7, 6, 6, 6 and 5 precursor members, respectively. The other conserved members have precursors greater than or equal to 2 are shown in **Figure 9**. The relative expression of miRNA genes is associated to their copy numbers or methylation or both (Aure et al., 2013); hence individually, a strong correlation with copy numbers cannot be predicted in complete miRNome. We have used SPSS for determining the bivariate Pearson correlation coefficient (PCC) between samples and the miRNA copy number with sig. (2-tailed) and bootstrap equal to 1000 at 95% confidence interval. We have observed significant but not highest positive correlation, i.e., PCC equal to 0.638 (*p*-value: 0.001) for highly conserved miRNA gene families (Axtell and Bowman, 2008) and for all 48 conserved miRNAs, the PCC is equal to 0.382 (*p*-value: 0.007). The correlation of all 176 predicted novel miRNAs is not significant with PCC equal to -0.52 (*p*-value: 0.494), suggesting their transient nature.

### Genomic Origins of miRNAs in *G. arboreum*

**FIGURE 10:**
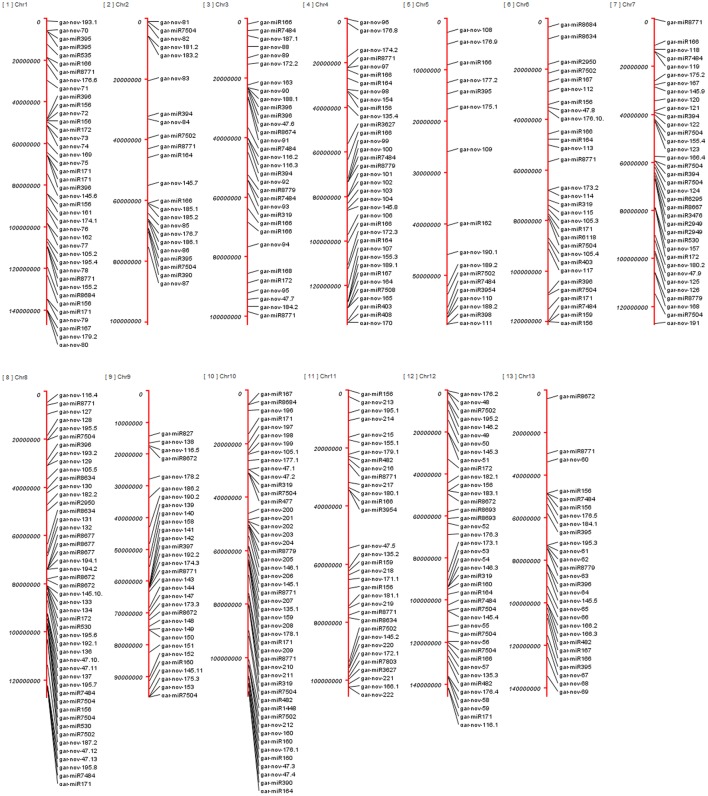
Genomic loci of miRNA genes in *G. arboreum.* The genomic origins of 446 miRNAs on different chromosomes of *G. arboreum* having conserved miRNAs labeled with their family names on the right side of red chromosomal vertical bars. The novel miRNAs are displayed as *gar-nov-ID*, whereas ID represents the ID given to each miRNA gene family, listed in Supplementary Table S1.

Altogether, 224 miRNA families originate from 446 different genomic origins due to varying number of copies and isoforms. All the chromosomes have nearly symmetrical number of miRNAs distributed over them, except chromosome 5; with only 17 miRNA genes were found. Chromosome 10 has the greatest number of miRNAs (49) predicted, followed by chr8 (48), chr12 (41), chr1 (40), chr4 (36), chr7 (36), chr11 (33), chr3 (32), chr9 (31), chr6 (30), chr13 (28), chr2 (23), and chr5 (17). Altogether, 19 miRNAs originated from 32 different intronic origins of different annotated protein coding genes including their isoforms and copy numbers, listed in Supplementary Table S5. These include conserved miRNA families gar-miR172, gar-miR395, gar-miR7484, gar-miR7502, gar-miR7504, gar-miR8634, gar-miR8674, gar-miR8779 and 11 novel miRNAs. Six out of seven copies of gar-miR7502 were originated from introns of different protein coding genes. Plants have less common phenomenon of clustered miRNA genes than animals (Zhang et al., 2006a; Voinnet, 2009). These clusters are mostly homologous family members of conserved miRNAs (Axtell and Bowman, 2008) and within a few kilobases in distance (Li and Mao, 2007). We have found 9 such clusters with a window size up to 8kb, including gar-miR171 and gar-miR7504 on chr1 and chr8, respectively, having intergenic distance of 2kb. The gar-miR2949 and gar-nov47 made clusters within 4kb distance on chr4 and chr8, respectively. The other clusters were made by gar-miR8677 and some species specific miRNAs within 8kb intergenic distances. Increasing the window size yields a few more clusters but they are less likely to be polycistronic. All of the clusters have two precursor members except miR8677, which contains three such genes in 8kb vicinity, as shown in Supplementary Table S6. The genomic origins of all of the predicted genes are shown in **Figure 10**, elaborating the distribution of miRNA genes over 13 chromosomes.

### Experimental Validation

**FIGURE 11:**
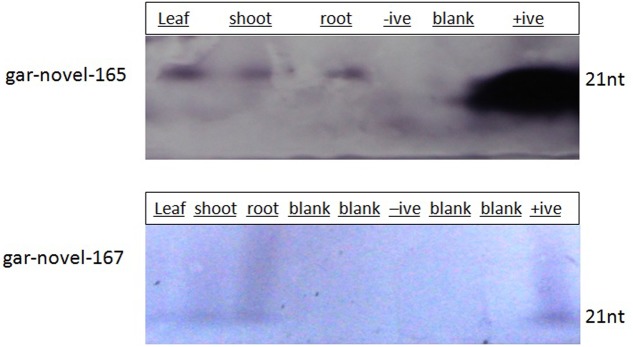
Experimental Validation of *G. arboreum* specific miRNAs. The bands show presence of a miRNA using RNA gel blot hybridization. The primers serve for positive control as well as length demarcation criteria whereas; wheat RNA served as negative control. For gar-novel-165, we have used GAGAGACATCTAAAGTACAAA and for gar-novel-167, CCCAAAGAGAACAATATCGAT as reverse complementary probes.

In order to validate the effectiveness of our bioinformatics pipeline, we chose two *G. arboreum* specific miRNAs (gar-novel-165: UUUGUACUUUAGAUGUCUCUC and gar-novel-167: AUCGAUAUUGUUCUCUUUGGG). These miRNAs originate from single genomic locus where gar-novel-165 is transcribed from positive strand and gar-novel-167 from negative strand. Their secondary structure is having MFEI value less than -1 whereas target of one of them (gar-novel-165) is not found. Both of these pre-miRNAs have good base pairing in the stem region MFE -76.7 and -91, respectively. The above selection criteria make these miRNAs ideal for validation and hence extrapolating the results for our *in silico* mapping based pipeline. We have extracted RNA from leaf, shoot and root tissues and leaves show relatively more expression level. Moreover, wheat leaves RNA was used as negative control and primers (complimentary sequence) as positive controls, as shown in **Figure 11**. This analysis showed that these miRNAs specifically expressed in *G. arboreum* and have different expression levels in different tissues.

## Discussion

MicroRNAs are master regulators of various cellular processes related to plant growth and various biotic and abiotic stresses by interacting with their targets at both transcriptional and post-transcriptional levels. They not only interact with protein coding gene mRNAs but also with several other RNAs called ceRNAs or miRNA sponges to regulate themselves as well as other ceRNAs (Le et al., 2016) and mRNAs. Animal miRNAs do not require perfect complementarily to their targets and interact with relatively more targets per miRNA whereas plant miRNAs are much more specific to relatively smaller target sets (Khraiwesh et al., 2012). Due to the extreme importance of these small endogenous molecules, bioinformatics tools and pipelines were developed to identify, characterize and analyze them in different biological contexts (Lei and Sun, 2014). Recently, HTS along with downstream bioinformatics analysis has proved quite successful in identifying low expression miRNA transcripts with precision and high accuracy at genome scale (Zhang et al., 2013). Hence, various new miRNAs have been added into the miRNA repository, mirBase (Griffiths-Jones et al., 2006) and several other repositories have also been developed for their annotations (Zhang et al., 2010; Szcześniak and Makałowska, 2014).

Many evolutionarily conserved and species specific or novel miRNA genes have been identified in various plants, especially in cotton species *G. hirsutum* and *G. raimondii*. However, there are 431 *de novo* predicted (Li et al., 2014) but only one experimentally verified miRNA (gar-miR2947) available in miRBase (release 21) for another important cotton species, *G. arboreum.* The estimated number of miRNAs present in its genome may be larger than this because of larger genome size (∼1.7Gb) as compared to *Arabidopsis thaliana* and *Oryza sativa*, for which 427 and 689 miRNAs have been reported to date (Griffiths-Jones et al., 2006). Although the direct economic impact of *G. arboreum* on cotton production is minor, its inherent superior resistance to various biotic and abiotic stresses and contribution of the AA genome to allopolyploid upland cotton justifies extensive genome scale biological understanding.

We have found 224 miRNA genes, out of which 48 are evolutionarily conserved across other plant species and 176 are less conserved or *G. arboreum* species specific. The conserved miRNA genes have varying copy numbers because of genomic segment duplications, inversions and deletions. The origins of these miRNAs were classified as intronic and intergenic but intergenic miRNAs may be originated from retrotransposons, which are major contributors to genome size variations in eukaryotes. The 24nt length is dominant in the predicted mature sequences, and genomic origin shows that only 32 originated from introns with the rest from intergenic regions. There are possible polycistronic miRNAs (Zhang et al., 2006a) as we have found some clusters within 2, 4, and 8 kb regions. The identification of polycistronic and miRNAs originating from retrotransposons require more detailed genome annotation, and may be a future subject of miRNA gene evolution in cotton. The reason for fewer conserved miRNA predictions is that we have selected stringent short-listing criteria (i.e., total mismatches equal to 3 and no gap). Moreover, the sample was taken only from leaf tissues and may be increased by sampling miRNAomes from multiple tissues and at different stages. The aligned transcripts have variation in sizes and alignment position to the stem-loop sequence because of inaccurate Dicer processing or trimming during preprocessing, the effect of which has been reduced by considering the most abundant transcript.

The *G. arboreum* miRNA target genes are involved in metabolic pathways including carbon, carbohydrate, energy, lipids, nucleotides, amino acids, glycan and Oxocarboxylic acid metabolism and others. Moreover, several targets in transcription regulation (RNA polymerase, transcription Factors, Spliceosome), translation, replication and repair mechanisms, signaling and various cellular processes like transport and catabolism, cell motility, growth and death and cellular community, plant pathogen interaction and aging have also been identified. The complex miRNA regulatory network, shown in **Figure 6**, illustrates that there are many relationships between regulating miRNAs and their target processes. The gar-miR156, gar-miR166, gar-miR3476 and gar-miR7502 not only play their role in signal transduction but gar-miR156 regulates cell growth and death as well as targets transcription factors (SBP) to regulate other genes and similarly gar-miR166, gar-miR3476 and gar-miR7502 miRNAs regulate different metabolic pathways. The origin of miRNA genes and their targets are functionally related with each other and may affect similar cellular processes or pathways. One copy of miR395 originates from Cotton_A_00405, which is an RNA-binding (RRM/RBD/RNP motifs) family protein and targets genes involved in sulfur, purine and selenocompound metabolism. Moreover, its targets genes are involved in ribosomal biogenesis pathway, enzymes like cellulose synthase, nucleotide-diphospho-sugar transferases, polyprenyl synthetase, terpenoid synthase and Solanesyl diphosphate synthase. One novel miRNA gar-novel67 originates from Cotton_A_06583, which is involved in the glycerol metabolic process andis an important modulator of root development, targets small GTPase superfamily and is also involved in ribosomal biogenesis. Members of miR8634 originated from a general transcription factor IIH2 and phosphorus-containing groups transferase protein whereas target proteins are involved in N-Glycan biosynthesis, carbon metabolism, glyoxylate and dicarboxylate metabolism and carbon fixation in photosynthetic organisms. Similarly, a member of gar-miR172 originates from a reverse transcriptase domain containing protein and targets HEAT, type2 or Armadillo-like helical domain containing proteins involved in mRNA surveillance and cell cycle pathways. The gar-miR7502, originated from intergenic regions as well as introns of 6 genes including gene involved in fructose and mannose metabolism and lipase and hydrolase activities and targets V-type ATPase, v0 complex protein and proteins involved in oxidative phosphorylation and involved in signal transduction, transport and cell growth and death pathways.

The conserved and *G. arboreum* specific miRNA targets are involved in several important biological processes related to plant growth and development, biotic and abiotic stress response and stress damage repair, cellular components and processes. The gar-miR166 exhibited the highest expression (Yin et al., 2012) level with reads per million (RPM) value up to 26912 and its target is homeobox-leucine zipper protein, which is a transcription factor that controls regulation of meristem development to promote lateral organ formation and may regulate procambial and vascular tissue formation or maintenance and vascular development in inflorescence stems and maintenance of developmental homeostasis (Peng et al., 2014; Xie et al., 2015) during stress conditions. The gar-miR156 targets Squamosa Promoter binding protein (SPL) which are master regulators of developmental processes, e.g., Panicle development and response to different biotic and abiotic stresses (Khraiwesh et al., 2012). The other important miRNAs like gar-miR160 targets Auxin Response Factors (ARF10, ARF17) which are key regulators of plants growth and developmental processes (Guilfoyle and Hagen, 2007), gar-miR164 targets NAC-domain containing proteins including cup-shaped cotyledon-1 which are involved in plant root and shoot development and in response to abiotic stresses like cold, abscisic acid, drought or high salt conditions (Huang et al., 2013). The gar-miR171 and gar-miR167 enhances abiotic stress tolerance by targeting GRAS transcription factors and cation efflux proteins. The GRAS play an important role in abiotic stress tolerance by repressing gibberellins signaling pathway (Shi et al., 2015).

In summary, we have identified conserved and *G. arboreum* specific miRNAs, using a genome wide approach by deep sequencing of sRNA. The genomic annotation can be further utilized in comparative and *G. arboreum* specific studies.

## Author Contributions

SM designed the experiment and isolated the RNA. PC, HG, and AHP helped in experimental setup. MF, IA, MKA, and SM did the analysis and finalized the manuscript.

## Conflict of Interest Statement

The authors declare that the research was conducted in the absence of any commercial or financial relationships that could be construed as a potential conflict of interest.

## Footnotes

1 http://www.lcsciences.com/

2 https://www.cottongen.org

3 http://www.kegg.jp/blastkoala/

4 http://pantherdb.org/panther/

5 http://www.genome.jp/kegg/pathway.html
